# Thyroid-Disrupting Chemicals: Interpreting Upstream Biomarkers of Adverse Outcomes

**DOI:** 10.1289/ehp.0800247

**Published:** 2009-02-12

**Authors:** Mark D. Miller, Kevin M. Crofton, Deborah C. Rice, R. Thomas Zoeller

**Affiliations:** 1 Office of Environmental Health Hazard Assessment, California Environmental Protection Agency, Oakland, California, USA; 2 Pediatric Environmental Health Specialty Unit, University of California at San Francisco, San Francisco, California, USA; 3 National Health and Environmental Effects Laboratory, Office of Research and Development, U.S. Environmental Protection Agency, Research Triangle Park, North Carolina, USA; 4 Maine Center for Disease Control, Augusta, Maine, USA; 5 Biology Department, Morrill Science Center, University of Massachusetts at Amherst, Amherst, Massachusetts, USA

**Keywords:** children’s health, endocrine disruption, hazard identification, risk assessment, science policy, thyroid hormone, toxicologic assessments

## Abstract

**Background:**

There is increasing evidence in humans and in experimental animals for a relationship between exposure to specific environmental chemicals and perturbations in levels of critically important thyroid hormones (THs). Identification and proper interpretation of these relationships are required for accurate assessment of risk to public health.

**Objectives:**

We review the role of TH in nervous system development and specific outcomes in adults, the impact of xenobiotics on thyroid signaling, the relationship between adverse outcomes of thyroid disruption and upstream causal biomarkers, and the societal implications of perturbations in thyroid signaling by xenobiotic chemicals.

**Data sources:**

We drew on an extensive body of epidemiologic, toxicologic, and mechanistic studies.

**Data synthesis:**

THs are critical for normal nervous system development, and decreased maternal TH levels are associated with adverse neuropsychological development in children. In adult humans, increased thyroid-stimulating hormone is associated with increased blood pressure and poorer blood lipid profiles, both risk factors for cardiovascular disease and death. These effects of thyroid suppression are observed even within the “normal” range for the population. Environmental chemicals may affect thyroid homeostasis by a number of mechanisms, and multiple chemicals have been identified that interfere with thyroid function by each of the identified mechanisms.

**Conclusions:**

Individuals are potentially vulnerable to adverse effects as a consequence of exposure to thyroid-disrupting chemicals. Any degree of thyroid disruption that affects TH levels on a population basis should be considered a biomarker of adverse outcomes, which may have important societal outcomes.

Recent epidemiologic studies have demonstrated significant relationships between circulating levels of thyroid hormones (THs) and exposures to environmental chemicals (Blount et al. 2006; Boas et al. 2006; Longnecker et al. 2003; Steinmaus et al. 2007). In controlled animal studies, environmental chemicals have been shown to cause a reduction in serum TH levels, also supporting a causal association (Boas et al. 2006; Brucker-Davis 1998; DeVito et al. 1999; Zoeller 2007). In this article we review the role of THs in development and adult life, the impact of xenobiotics on thyroid status, the relationships between adverse outcomes of thyroid disruption and upstream causal biomarkers, and the societal implications of perturbations in THs by xenobiotic chemicals.

## The Role of THs in Development

THs include both thyroxine (T_4_) and triiodo-thyronine (T_3_). The independent regulation of circulating levels of these two forms of TH is complex, but in this review we refer generally to both forms as TH. THs are evolutionarily conserved molecules present in all extant vertebrates and some invertebrates (Heyland and Moroz 2005). Molecular signaling pathways regulated by these hormones affect development, energy balance, and metabolism in all taxonomic groups. For example, TH induces metamorphosis in the sand dollar (Heyland et al. 2004), flounder (Yamano et al. 1994), and frogs (Buchholz et al. 2005), and TH is essential for development in birds (McNabb 2006) and mammals (Zoeller and Rovet 2004). In humans, TH is important for normal development of brain (Bernal 2007; Oerbeck et al. 2007), lungs (Bizzarro and Gross 2004; van Tuyl et al. 2004), heart (Danzi et al. 2005; Grover et al. 2005; Stoykov et al. 2006), and other organs. Likewise, the mechanism(s) by which THs exert their actions through nuclear receptors that influence gene expression is highly conserved across the vertebrate taxa (Bertrand et al. 2004; Buchholz et al. 2006; Whitfield et al. 1999).

**Figure 1:**
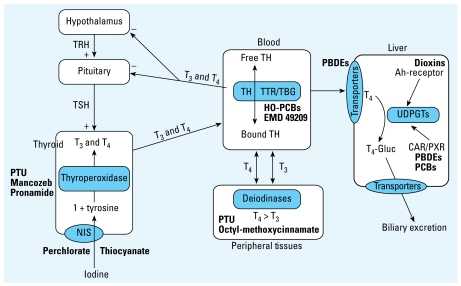
TH control pathways and sites of disruption by xenobiotic chemicals. Abbreviations: Gluc, glucose; HO-PCBs, hydroxyl-PCBs; NIS, sodium/iodide symporter; PBDE, polybrominated diphenyl ether; PTU, propylthiouracil; T_4_-Gluc, T_4_-glucuronide; TBG, thyroid-binding globulin; TRH, thyrotropin-releasing hormone; TSH, thyroid-stimulating hormone; TTR, transthyretin; UDPGT, uridine diphosphate glucuronyl-transferase. Sites or processes where xenobiotics are known or hypothesized to act as TDCs are indicated in the boxes and ovals. Xenobiotics that block, inhibit, or up -regulate these processes are shown in bold (modified from Crofton 2008).

The regulation of serum TH levels and of TH action in various tissues involves a complex interplay of physiologic processes. Thyroid function depends on iodine uptake, TH synthesis and storage in the thyroid gland, stimulated release of hormone into and transport through the circulation, hypothalamic/pituitary control of TH synthesis, cellular TH transporters, tissue-specific TH deiodination, and degradation of THs by catabolic hepatic enzymes (Figure 1). Given the key role of TH for normal development and physiologic function in all vertebrates, it is important to identify environmental factors that may adversely affect thyroid function and/or TH signaling and to evaluate their ability to adversely affect public health (Brucker-Davis 1998). In addition, because of the highly conserved nature of TH chemistry, synthesis, signaling, and regulation, environmental factors that affect thyroid function or TH signaling in one species may well affect thyroid function or TH signaling in others—including humans.

### THs and nervous system development

It is becoming clear that, although somatic and brain growth retardation occur with severe TH insufficiency, moderate or even transient TH insufficiency can cause specific developmental defects in rodents (Auso et al. 2004; Crofton 2004; Crofton et al. 2000; Goldey et al. 1995a, 1995b; Goodman and Gilbert 2007; Morreale de Escobar 2003) and in humans (Haddow 2005; Haddow et al. 1999; Kooistra et al. 2006; Oerbeck et al. 2003, 2007; Pop et al. 1999, 2003; Pop and Vulsma 2005). Small differences (~25%) in point-estimates of maternal T_4_ during the early fetal period are associated with adverse outcomes (e.g., reduced IQ scores), even though these deficits do not constitute clinical hypothyroidism (Haddow et al. 2002; Morreale de Escobar et al. 2000). However, in a hallmark study by Bongers-Schokking et al. (2000), the Mental Development Index of children with congenital hypothyroidism was affected by the age of onset of treatment, rather than the serum free T_4_ concentration after treatment. Thus, the degree of TH insufficiency is not the only variable affecting human development; the duration of the insufficiency and the developmental timing of the insufficiency are also important and may vary by species, presenting a challenge for hazard assessment.

Experimental work in animals provides strong support for the hypothesis that moderate TH insufficiency can alter development in rodents. Integrating data over a series of studies, a decrease in serum total T_4_ by 50% during the critical period for cochlear development was associated with a permanent hearing loss in adult offspring (Crofton 2004). Auso et al. (2004) found that less than a 30% decrease in serum total T_4_ in dams, for only 3 days, was associated with structural abnormalities in the brains of their offspring. An average decrease in serum total T_4_ of only 28% in 2-week-old pups given low doses of propylthiouracil was associated with marked reduction in cell density of the corpus callosum (Sharlin et al. 2008). Gilbert and Sui (2008) found that a 28% reduction in circulating levels of T_4_ in rat dams produced significant adverse effects on synaptic function of the adult offspring despite no detected change in serum T_4_ levels in the pups after birth. Thus, these experimental findings confirm what has been observed in humans: small, even transient, decreases in serum total T_4_ are associated with altered brain development.

## TH Effects in Other Organ Systems and Adults

**Figure 2:**
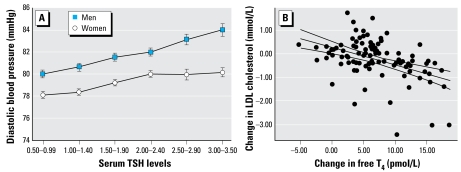
Population changes in diastolic blood pressure (*A*) and cholesterol (*B*) in relation to serum TSH or free T_4_, respectively. (*A*) Diastolic blood pressure in men and women are significantly correlated with serum TSH within the normal reference range for TSH, indicating that as serum T_4_ declines, diastolic blood pressure increases. (*B*) Serum cholesterol is negatively associated with serum free T_4_. An increase in free T_4_ by 5, 10, or 15 pmol/L would reduce LDL cholesterol by 0.13, 0.53, and 0.93 mmol/L, respectively. The data are redrawn with permission from Asvold (2007b; *A*) and from Razvi (2007; *B*) (Copyrights 2007, The Endocrine Society).

It is important to recognize that TH concentrations are correlated with adverse effects in organ systems other than the nervous system, including the cardiovascular system and control of serum lipids (Asvold et al. 2007a; Biondi et al. 2005; Osman et al. 2001), pulmonary system (Krude et al. 2002; Lei et al. 2003; Mendelson and Boggaram 1991), and kidney. Total cholesterol, low-density lipoproteins (LDL), non-high-density lipoproteins (non-HDL), and triglycerides increased linearly with increasing thyroid-stimulating hormone (TSH), and HDL decreased consistently with increasing TSH across normal reference ranges without evidence of any threshold effect (Asvold et al. 2007b). Similar trends in lipid profiles were identified across clinical categories from hypothyroid to euthyroid to hyperthyroid individuals (Canaris et al. 2000). Within the reference ranges for TSH, there was a linear positive association between TSH and both systolic and diastolic blood pressure (Asvold et al. 2007b) (Figure 2). Intimal medial thickness, a measure of atherosclerosis and predictive of coronary vascular disease and stroke, was inversely related to free T_4_ after controlling for lipids, clinical factors, and thyroid autoantibodies (Dullaart et al. 2007). Some of these adverse effects were ameliorated by treatment with T_4_. Not surprisingly, deficits in thyroid homeostasis were associated with cardiovascular risk in multiple epidemiologic studies. A meta-analysis of 14 epidemiologic studies (Rodondi et al. 2006) found an overall increase in risk of coronary heart disease of > 65% in those with subclinical hypothyroidism (elevation in TSH with normal T_4_). A higher relative risk was noted in those studies that adjusted for most cardiovascular risk factors, suggesting that confounding was not responsible for these effects. Treatment with L-T_4_ of patients with subclinical hypothyroidism resulted in improvements in cardiovascular risk factors, including total cholesterol and endothelial function (flow-mediated dilatation) (Razvi et al. 2007). Michalopoulou et al. (1998) found that treatment with T_4_ of hypercholes-terolemic individuals who have “high normal” TSH values significantly reduced both total and LDL cholesterol, additionally supporting a causal association. In addition, environmental exposure to the thyroid-disrupting chemical (TDC) polychlorinated biphenyls (PCBs) had an inverse association with T_3_ in men (Meeker et al. 2007) and was associated with both unfavorable lipid profiles and self-reported cardiovascular disease in men and women (Goncharov et al. 2008). Therefore, epidemiologic as well as mechanistic and therapeutic evidence substantiates the concern that TDCs may adversely affect cardiovascular risk in humans by reducing serum T_4_.

## Impact of Xenobiotics on TH Signaling

TDCs are broadly defined as xenobiotics that interfere with TH signaling. These can include chemicals that alter the structure or function of the thyroid gland (e.g., perchlorate and methimazole), alter binding of hormones to thyroid receptors (e.g., bisphenol A, PCBs, and polybrominated diphenyl ethers), or alter regulatory enzymes associated with TH synthesis (e.g., propylthiouracil) (Crofton et al. 2005). A number of extrathyroidal mechanisms affect TH levels by altering binding to hormone transport proteins (e.g., hydroxyl-PCBs), hepatic clearance (e.g., PCBs, triclosan), inhibition of deiodination to T_3_ (e.g., FD&C red dye number 3), and receptor agonism/antagonism (e.g., tetrabromobisphenol A). The downstream consequences of these effects are to alter TH-directed transcription either directly or via changes in circulating or tissue concentrations of THs. Several uncertainties complicate basic risk assessment approaches when assessing the hazards of TDCs. These include defining the biomarkers used for assessing hazard, defining the magnitude of change in the biomarker(s) that reliably predict downstream adverse outcomes, intraspecies extrapolation that is hampered by a lack of mechanistic and dose response data, and predicting the effects of real life exposures to low-level mixtures of xenobiotics that contain components that individually have vastly different kinetic and dynamic properties.

Several specific chemicals were shown to bind to TH receptors (TRs) (Zoeller 2005, 2007). This has important implications because there is good evidence that different effects of TH in the developing brain are mediated by different TR isoforms (Bernal 2007). There are two different classes of TRs (TRα and TRβ), and different chemicals can selectively interact with various isoforms. Thus, these chemicals will likely produce a mosaic of effects on TH signaling in the developing brain and may do so without affecting circulating levels of TH. It also may be challenging to develop high-throughput *in vitro* screens for TR binding because many of these screens use only the ligand-binding domain of the receptor, and there is some evidence that environmental chemicals can bind to an allosteric site on the DNA binding domain of the TR (Miyazaki et al. 2008).

**Figure 3:**
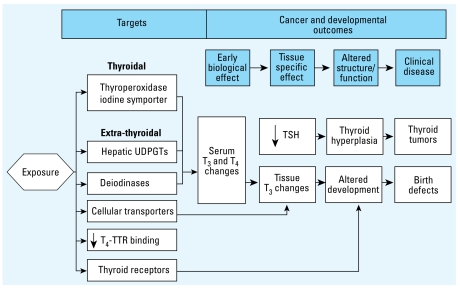
A combined mode-of-action model for the effects of TDCs on cancer and developmental outcomes. Abbreviations: TTR, transthyretin; UDPGT, uridine diphosphate glucuronyltransferase. Mixture models are needed to better predict effects of mixtures containing xenobiotics that affect multiple targets with common downstream effects (modified from Crofton and Zoeller 2005; U.S. EPA 2002).

The variety of mechanisms by which TDCs alter TH signaling (Table 1) provide a number of biomarkers that could be used in assessing hazard. These include molecular targets, which could be chemical-class specific, and downstream consequences, such as serum TH concentrations, brain morphology or biochemistry, or behavior. These changes may be either directly or indirectly related to TH action (Figure 3). Accurately and thoroughly assessing the health risks of thyroid disruption by environmental xenobiotics will require an improved understanding of how divergent mechanisms alter the relationship between serum THs and consequent adverse impacts on health.

The most commonly used biomarker of effect for TDC exposure is serum total T_4_ concentrations (DeVito et al. 1999; Zoeller et al. 2007). Although TSH is a well-accepted biomarker for hypothyroidism, a number of xenobiotics alter circulating TH levels but do not change TSH (DeVito et al. 1999). Therefore, it is central to risk assessment to understand the relationship between perturbations in circulating concentrations of T_4_ and adverse effects. In addition, it is important to test the hypothesis that changes in circulating concentrations of T_4_ represent a common pathway by which adverse outcomes are produced. This hypothesis is consistent with the accepted role of circulating concentrations of T_4_ in defining thyroid disease (Brabant et al. 2006). Many kinds of adverse effects are associated with either TH excess or insufficiency, depending on the timing, severity, and duration of the perturbation. Although the pattern of effects may differ, changes in serum TH are predictive of downstream adverse outcomes.

**Figure 4:**
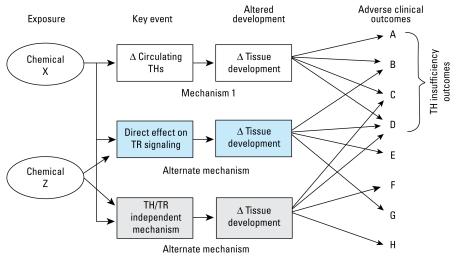
Diagnostic relationships between upstream biomarkers and adverse outcomes.

Upstream biomarkers of TDC exposure are predictive of adverse effects if the mechanisms of action are well characterized. Mechanism 1 in Figure 4 illustrates this point: alterations in circulating THs during development are predictive of adverse neurodevelopmental outcomes. This concept has been known for decades and is the basis for newborn TH screening (Rose et al. 2006). These adverse consequences are well documented in animals for xenobiotics that alter circulating levels of TH (Crofton and Zoeller 2005; Zoeller and Crofton 2005).

## Cross-Species Extrapolation

Although interspecies extrapolation of adverse effects of TDCs requires careful consideration, there are many situations in which the effects of a chemical in one species are similar to those in another, including in humans. For example, perchlorate competitively inhibits iodine uptake into the thyroid gland, with subsequent decreases in TH synthesis and declines in circulating TH concentrations (Wolff 1998). The kinetics for perchlorate inhibition of iodine uptake in humans and rats are extremely similar [U.S. Environmental Protection Agency (EPA) 2002], indicating the homologous nature of the initial toxic event. However, species differences in the relationship between changes in serum total T_4_ and downstream adverse effects, perhaps mediated by differences in kinetics such as tissue TH concentrations and the sensitivity of specific developmental outcomes to low T_4_, cannot be ruled out at this time (National Research Council 2005).

For some TDCs, there may be little data to support cross-species extrapolation (Crofton 2004). Both *in vivo* and *in vitro* studies suggest that PCBs activate the pregnane X receptor (PXR) in rodents, which leads to up-regulation of hepatic catabolic enzymes and subsequent declines in circulating concentrations of T_4_ (Schuetz et al. 1998). The steroid X receptor (SXR) is the human equivalent for rodent PXR (Blumberg et al. 1998), and there are species differences between PXR and SXR: Rodent PXR is activated by pregnenolone-16α-carbonitrile (PCN), but not by rifampicin, whereas human SXR is activated by rifampicin but not by PCN (Kliewer et al. 2002). In addition, *in vitro* data suggest that high concentrations of PCB-153 act as an antagonist at the human SXR (Tabb et al. 2004). As well, species differences in circulatory transport proteins (e.g., transthyretin and thyroid-binding globulin) complicate extrapolation from animals to humans (Capen 1997; Hill et al. 1998). Thus, species differences in the expression or structure of specific functional proteins (e.g., receptors and enzymes) may at times affect the toxicity of specific compounds in different species.

## Mixtures

Evaluating the potential for additive or synergistic (i.e., greater than additive) effects resulting from exposure to mixtures or environmental xenobiotics presents challenges for the assessment of endocrine disruptors (Daston et al. 2003). Additivity for mixtures of chemicals with a similar target is now a default assumption for some classes of chemicals (U.S. EPA 2000). A variety of predictive models are available for use with mixtures of similarly acting chemicals (Feron and Groten 2002; Kroes et al. 2005; Mumtaz et al. 1993; Teuschler 2007; U.S. EPA 2000). For example, the toxic equivalents methodology predicts the cumulative effects of aryl hydrocarbon receptor (AhR) agonists using dose addition (Haws et al. 2006; Van den Berg et al. 2006). However, these models may not predict effects of mixtures containing chemicals with multiple mechanisms of action (e.g., synthesis inhibitors, low dietary iodine, hepatic catabolism). The small number of studies reporting effects of mixtures of TDCs lack, either by study design or statistical approach, the ability to test for additivity (Desaulniers et al. 2003; Khan et al. 2005; McLanahan et al. 2007; Wade et al. 2002). The use of rigorous statistical models is critical for testing hypotheses of effect or dose addition and determining whether antagonism or synergism exists (Feron and Groten 2002; Hertzberg and Teuschler 2002; LeBlanc and Olmstead 2004).

**Figure 5:**
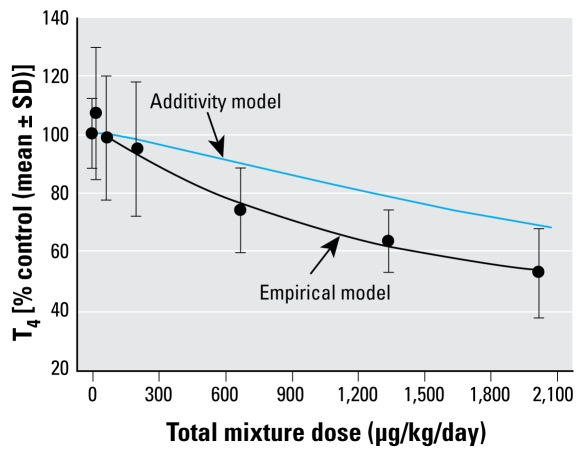
The predicted and empirical effects of a mixture of dioxins, furans, and PCBs on serum total T_4_ in rats. Predicted outcomes (additivity model) were generated using a single chemical-required additivity model. Empirical results (empirical model) showed a small but significant departure from dose additivity at the three highest mixture doses, whereas the remaining lower mixture doses were not significantly different than that predicted by additivity (modified from Crofton et al. 2005).

Crofton et al. (2005) tested a mixture of 18 TDCs (dioxins, dibenzofurans, and PCBs) for effects on serum T_4_. These chemicals were each known to decrease circulating concentrations of T_4_ (Craft et al. 2002; Crofton et al. 2005; Khan and Hansen 2003; McLanahan et al. 2007). The mechanisms by which these chemicals alter THs involve up-regulation of hepatic catabolic enzymes (e.g., uridine diphosphate glucuronosyltransferases). 2,3,7,8-Tetrachlorodibenzo-*p*-dioxin (TCDD), dibenzofurans, and dioxin-like PCBs activate a network of phase II and III proteins via binding of the AhR (Schrenk 1998). The non-dioxin-like PCBs activate a slightly different set of enzymes (and possibly transporters) via binding to PXR and the constitutive androstane receptor (CAR) (Kretschmer and Baldwin 2005; Schuetz et al. 1998). These differences in mechanisms of action (i.e., AhR agonists and CAR/PXR agonists) suggest that dose addition theory would not predict the effects of the mixture. A “flexible single-chemical-required” method (Casey et al. 2004; Gennings et al. 2002) demonstrated no deviation from dose additivity at the lowest doses of the mixture but a greater-than-additive effect at the highest mixtures doses (Figure 5). At high doses the dose-additivity model underpredicted the empirical effects by 2- to 3-fold but worked well at lower doses typical of environmental exposures.

Future work is needed to improve the ability of mixtures models to account for the homeostatic processes that are activated by changes in both tissue and serum TH concentrations. The paucity of data in this area makes it difficult to determine whether these models will accurately predict changes in common downstream adverse outcomes after exposure to complex mixtures of chemicals that act on multiple upstream targets. Indeed, the effects of the complex mixtures will likely depend on the interaction of both kinetic and dynamic factors. Increasingly, it may become possible to identify interactions of chemicals in population-based biomonitoring databases. For example, sizable subpopulations for whom the relationship between perchlorate exposure and serum T_4_ concentrations are modified by coexposure to thiocyanate, nutrition (iodide consumption), and behavior (smoking) have been identified using the National Health and Nutrition Examination Survey database (Blount et al. 2006; Steinmaus et al. 2007). Because additivity or synergy of TDCs with different mechanisms of action has been demonstrated, as noted above, a broad approach to cumulative risk that would account for these interactions seems appropriate. This is particularly true considering the limitations of current modeling methodologies.

## Causality

A critical issue affecting the interpretation of upstream events is the relationship between biomarkers captured in clinical or animal studies and specific adverse outcomes. Studies involving upstream biomarkers are most useful when these biomarkers have been causally linked to downstream adverse outcomes. For example, interpreting studies of perchlorate and T_4_ are relatively straightforward because the only known toxic effect of perchlorate is interference with thyroid function (National Research Council 2005); thus, any effects of perchlorate on the nervous system are necessarily interpreted to be subsequent to a reduction in serum THs.

Difficulties can arise when attempting to predict changes in upstream biomarkers based on adverse outcomes. For example, if the adverse outcome(s) of a specific toxicant or mixture is caused by more than one mechanism, then individual downstream outcomes (i.e., “effects”) are not diagnostic of upstream events, and causative links between a known exposure and outcome are difficult to discern. Figure 4 illustrates this by the alternative mechanisms activated by chemical X that may cause similar adverse outcomes. Indeed, some of these adverse outcomes may be caused by exposure to other chemicals (chemical Z). A key to using adverse outcomes in these cases is the use of patterns of outcomes that may be diagnostic.

PCBs offer a good example of the problems associated with inferring upstream changes in THs as the causative agent of downstream neurotoxic outcomes. PCBs produce changes in a number of behavioral domains in humans and animals (Rice 2000; Schantz et al. 2003). They also affect multiple neurochemical pathways (Kodavanti et al. 1993; Kodavanti and Ward 1998; Seegal 1996; Seegal et al. 1991) in addition to TH (Crofton and Zoeller 2005). Although changes in THs during development predict specific behavioral changes, effects of PCBs on some specific tasks in animals or outcomes in epidemiologic studies may not necessarily be attributable to changes in THs.

Another example of the difficulty in linking serum TH to adverse outcomes is provided by the recent observation in humans of an abnormal TH profile in boys with a genetic mutation in the T_3_-specific transporter mono-carboxylate anion transporter 8 (*MCT8*). In all cases, serum T_3_ is elevated, but serum T_4_, free T_4_, and TSH may be low, normal, or elevated (Jansen et al. 2007). Thus, the elevated serum T_3_ appears to be a biomarker of the MCT8 mutation among the patients evaluated, although it is not the only mechanism by which T_3_ can become elevated. In addition, all of the boys evaluated presented with severe psychomotor deficits, but it is unlikely that the elevated serum T_3_ itself was the root cause of their condition. Thus, environmental factors that influence T_3_ transport through MCT8 may represent a situation in which the profile of serum TH hormones is perturbed in ways that are not immediately recognizable as due to an endocrine disruptor, but may signal that adverse effects occur through a mechanism that interferes with TH signaling.

Recognition of the role of “critical windows of exposure” in characterizing causal relationships between toxicant effects on serum THs and downstream adverse effects is critical. Specifically, the role of TH in brain development changes as development proceeds (Zoeller and Rovet 2004). Therefore, to establish a causal role of toxicant-induced low TH in the mechanism of neurotoxicity, it is important to show that T_4_ replacement can reverse the effects of toxicant. However, it is important to be cognizant of the relevant “windows” of vulnerability in the design of these experiments. For example, the impact of TH disruption on the development of auditory function in rats correlates well with circulating T_4_ levels during the second postnatal week (Crofton 2004). This is entirely consistent with the known role of THs in auditory development (Uziel et al. 1981), the critical postnatal ontogeny of auditory function (Rubel 1978), and the pharmacokinetics of the chemicals tested (Crofton and Zoeller 2005). In addition, this correlation establishes a prognostic power of early postnatal T_4_ for adverse consequence of developmental exposure to TDCs in rats (Crofton 2004). An understanding of the role of THs in development, coupled with hormone level measurement during the critical window, allows the establishment of a developmental mode of action that assigns a key causative role to TH disruption in the adverse outcome (Figure 4).

Studies designed to test for associations between toxicant exposures and circulating levels of TH in humans require careful consideration of confounding variables. For example, blood levels of TH vary among individuals (Andersen et al. 2002, 2003), which will affect the number of samples required for such a study to be sufficiently powered to identify associations of interest. In the case of newborn TH levels, a number of maternal, infant, and delivery factors influence TH levels in cord blood and in infant serum (Herbstman et al. 2008), and these must be carefully considered when attempting to identify associations between toxicant exposures and serum TH levels. A good recent example is that of Herbstman et al. (2008), who showed that PCB measures in cord blood were associated with circulating levels of TH only in those babies born via an unassisted vaginal delivery. Thus, these confounding variables may explain the studies in which PCB body burden has not been found to be associated with THs.

## Sensitive Populations

There may be individuals within the general population who are more at risk than others (i.e., sensitive subpopulations). For example, because pregnancy causes an increased demand on the thyroid gland, pregnant women may be particularly sensitive to specific kinds of toxicants that produce an additional burden on the thyroid gland, such as perchlorate, or chemicals that activate liver metabolism of T_4_. Women in general appear to be more sensitive to the adverse effects of perchlorate (Blount et al. 2006), although it is not clear why. An estimated 7.3% of the U.S. population either have self-reported hypothyroidism or take thyroid medication, and three-quarters of these are women (Aoki et al. 2007). More than 17% of those > 12 years of age report taking medications known to alter TH levels (e.g., estrogen, lithium, androgens). Those 50–79 and ≥ 80 years of age have a 2-fold and 5-fold increased risk of hypothyroidism, respectively, compared with those 12–49 years of age (Aoki et al. 2007). These are examples of large sub-populations at risk with any additional exposures that affect thyroid homeostasis.

**Figure 6:**
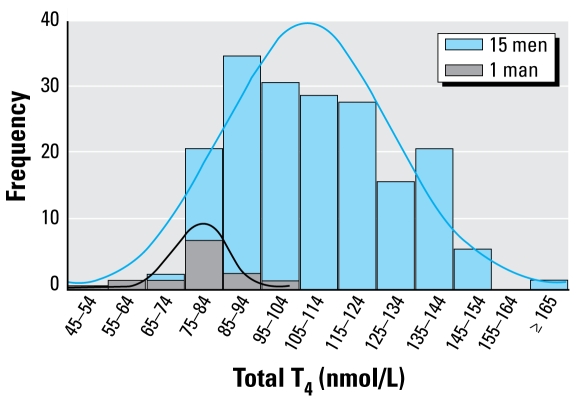
Individual versus population reference range for T_4_: the distribution of 12 monthly measurements for 15 men compared with one individual. The distribution width for the individual is approximately one-half that of the group [adapted from Andersen et al. (2002); copyright 2002, The Endocrine Society].

The set-point around which THs are regulated is very individualistic (Andersen et al. 2002, 2003), and differences between individuals in their set-point is largely determined by genetics (Hansen et al. 2004). Epidemiologic studies have identified elevated risk of cardiovascular disease in patients with subclinical hypothyroidism, characterized by elevated TSH with normal T_4_. Many studies identify that TDCs are associated with decreases in T_4_ but not elevations in TSH. However, the low level of interference with thyroid homeostasis seen in subclinical hypothyroidism and with TDCs may be equivalent, suggesting that elevated risk of cardiovascular disease should be considered possible from exposure to TDCs. The variance in serum T_3_, T_4_, and TSH in individuals is about half of the range of population variance, known as the “reference range,” as shown for T_4_ in Figure 6 (Andersen et al. 2002). Therefore, a value within standard “normals” is not necessarily normal for the individual, and an elevated TSH (which responds with a logarithmically amplified variation to minor changes in T_3_ and T_4_) should be interpreted as indicating that serum T_3_ and T_4_ levels are not normal for the individual (Andersen et al. 2002). Thus, it is highly likely that unidentified subpopulations exist that have particular sensitivity to thyroid disruption. The ability of epidemiologic studies to identify associations between thyroid disruptors and cardiovascular (or other) outcomes may be diminished as a result of failure to recognize risk in individuals who may have T_4_ levels in the normal population range but below their own normal individual range. Therefore, any exposure that would result in altered TH homeostasis in a population should be considered an adverse effect.

## Societal Burden

The burden to society of even small changes in function should not be dismissed or underestimated. The consequences of developmental lead exposure provide an informative example of the effects of a small shift in the IQ of a population. Lead exposure has been widespread in the United States, although blood lead concentrations decreased from a mean toddler blood lead of 15 μg/dL to < 2 μg/dL over the past four decades with the introduction of nonleaded gasoline and other measures ( Centers for Disease Control and Prevention 2007). A mean toddler blood lead of 15 μg/dL would be expected to decrease population IQ by ≥ 5 points (Lanphear et al. 2005). Although the consequences of a 5-point decrease in an individual’s IQ may be difficult to discern, the impact of this 5% shift at the tails results in a 57% national increase in those classified as mentally retarded (IQ < 70) and a concomitant decrease in individuals considered gifted (IQ > 130) (Schettler 2001; Weiss 1997).

Small decrements in maternal T_4_ or free T_4_ during the first trimester are associated with impaired neuropsychological development in the child (Haddow 2005; Haddow et al. 1999; Oerbeck et al. 2003, 2007; Pop et al. 1999, 2003; Pop and Vulsma 2005). However, children born to women with moderately low TH identified in these studies largely fall within the lower portion of the normal range for measures of neuropsychological function. Although they have lower IQ as a population, their individual IQ is in the normal range (Haddow 2005; Haddow et al. 1999).

**Figure 7:**
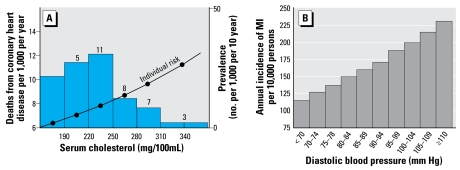
Individual risk and mortality associated with MI. ( *A* ) Individual risk and prevalence for MI associated with increased serum cholesterol levels. The number above each bar represents estimate of attributable deaths per 1,000 per 10 years. Note that individual risk increases linearly (including within the range of values considered normal) but that most deaths attributable to increased cholesterol levels occur in the lower range, because this represents a greater proportion of the population (adapted from Rose 1981; with permission from the BMJ Publishing Group). (*B*) Death from MI associated with increased diastolic blood pressure in males 45–74 (age-adjusted rate) (adapted from U.S. EPA 1985).

The cardiovascular consequences of disruption of thyroid homeostasis also potentially affect a large portion of the adult population. As noted above, there is a linear association between TSH (including through the normal reference range) and both blood pressure and cholesterol (Asvold et al. 2007a, 2007b). The magnitude of these changes associated with changes in THs would be considered to confer minimal risk to an individual, even though the individual risk of myocardial infarction (MI) and death from MI increases linearly for increased systolic and diastolic blood pressure (U.S. EPA 1985) and serum cholesterol (Rose 1981) (Figure 7). There is an important distinction that needs to be recognized, however: the difference between individual (relative) risk and population-attributable risk. Typically, the medical community assigns specific values for blood pressure and cholesterol as “high” or “borderline” to advise individuals on individual health risk. However, as illustrated in Figure 7, most of the morbidity in the population as a whole is associated with lower rather than higher levels, because a higher percentage of the population falls within the low to moderate range (Rose 1985; Rose and Day 1990).

The population-attributable risk can be used to monetize the societal burden of exposure to chemicals that affect thyroid function. For example, the U.S. EPA estimated the effects of lead, which is associated with increases in both systolic and diastolic blood pressure, on cardiovascular function (U.S. EPA 1985). The monetary burden of lost IQ associated with lead or methylmercury exposure has also been estimated at billions of dollars per year (Landrigan and Garg 2002; Trasande et al. 2006). Similar estimations could be made for the burden of exposure to chemicals that decrease THs and result in IQ deficits or increased incidence of cardiovascular disease. It is important to recognize that these outcomes are not only relevant if “abnormal” (e.g., mental retardation, clinically defined high blood pressure, or high cholesterol) but also relevant to outcomes in the “normal” range. Therefore, it is extremely important not to confuse the goal of minimizing population risk with arguments focused on individual relative risk.

## Conclusions

Two conclusions follow from the recognition that thyroid dysfunction affects multiple end points and that population-attributable risk is greater at levels associated with lower individual risk. First, from fetal life through old age, people are potentially vulnerable to adverse health effects as a consequence of exposure to TDCs. Second, any degree of thyroid disruption that lowers TH levels on a population basis should be considered a biomarker of increased risk of adverse outcomes. Because TH insufficiency in both humans and experimental animals results in serious neurodevelopmental and cardiovascular effects with large societal costs, chemicals with the ability to affect thyroid homeostasis should be carefully evaluated for potential population impacts. Finally, considering the complexity of the regulatory mechanisms affecting TH signaling and the variety of known TDCs that affect the thyroid system at different points of regulation, it will be essential to incorporate new information in human risk assessment strategies as it becomes available.

## Figures and Tables

**Table 1:** Classes, mechanisms of action, and effects of TDCs on TH homeostasis.

Class	Mechanism	Effect on THs	Chemical	References
Iodine transport	Competition/block of sodium/iodide symporter	Decreased thyroidal synthesis of T_3_ and T_4_	Perchlorate, chlorate, bromated nitrates, thiocyanate	Tonacchera et al. 2004; Van Sande et al. 2003; Wolff 1998

Synthesis inhibitors	Inhibition of thyroid peroxidase	Decreased thyroidal synthesis of T_3_ and T_4_	Methimazole, propylthiourea, amitrole mancozeb, soy isoflavones, benzophenone 2,1-methyl-3- propylimidazole-2-thione	Biegel et al. 1995; Capen 1997; Doerge and Sheehan 2002; Hurley 1998; Schmutzler et al. 2007

Transport disruption	Altered binding to serum transport proteins	Unknown	Hydroxyl-PCBs, EMD 49209, pentachlorophenol	Lans et al. 1993; Schroder-van der Elst et al. 1997; van den Berg 1990

Enhanced hepatic catabolism	Up-regulation of glucuronylsyltransferases or sulfotransferases (via CAR/PXR or AhR)	Increased biliary elimination of T_3_, T_4_	Acetochlor, phenobarbital, 3-methylcolanthrene, PCBs, 1-methyl-3-propylimidazole-2-thione	Biegel et al. 1995; Brucker-Davis 1998; Hood and Klaassen 2000; Hurley 1998; Liu and Klaassen 1996

Enhanced cellular transport	Up-regulation of organic anion-transporting polypeptides or MCT transporters via CAR/PXR or AhR	Increased biliary elimination of T_3_, T_4_	1,4-Bis[2-(3,5-dichloropyridyloxy)] benzene, PCN, TCDD, rifampicin, phenobarbital, oltipraz	Guo et al. 2002; Jigorel et al. 2006; Petrick and Klaassen 2007; Staudinger et al. 2001

Sulfotransferases	Inhibition of sulfotransferases	Decrease sulfation of THs	Hydroxy-PCBs, triclosan, pentachlorophenol	Schuur et al. 1998; Wang et al. 2004; Wang and James 2006

Deiodinases	Inhibition or up-regulation of deiodinases	Decreased peripheral synthesis of T_3_	FD&C red dye no. 3, propylthiouracil, PCB, octylmethoxycinnamate	Capen 1998; Klammer et al. 2007; Morse et al. 1993; Visser et al. 1979

TR agonists and antagonists	Direct or indirect alterations in TR–T_3_ response element binding	Altered activation of TH-dependent gene transcription	Tetrabromobisphenol A, bisphenol A, hydroxy-PCBs	Gauger et al. 2004; Kitamura et al. 2005; Moriyama et al. 2002

Abbreviations: Ahr, aryl hydrocarbon receptor; CAR, constitutive androstane receptor; FD&C red dye no. 3, Food, Drug and Cosmetics red dye no. 3; PCN, pregnenolone-16a-carbonitrile; PXR, pregnane X receptor. Modified from Crofton (2008).
